# More Fixation, Better Outcome? Evaluating the Role of Additional Acromioclavicular Ligament Reconstruction in AC Joint Injuries: A Multicenter Analysis

**DOI:** 10.3390/jcm14248679

**Published:** 2025-12-08

**Authors:** Gregor Wollner, Samuel Luisi, Florian Hruska, Florian Pengg, Felix R. M. Koenig, Gustav Timmel, Michael Osti, Christian Bach, Thomas Haider

**Affiliations:** 1 Department of Orthopedics and Trauma-Surgery, Medical University of Vienna, 1090 Vienna, Austria; 2 Department of Orthopedics and Traumatology, Klinik Floridsdorf, 1210 Vienna, Austria; 3 Department of Biomedical Imaging and Image-Guided Therapy, Medical University of Vienna, 1090 Vienna, Austria

**Keywords:** AC joint, acromioclavicular joint injury, acromioclavicular joint fixation, trauma, shoulder-surgery

## Abstract

**Background/Objectives:** Acromioclavicular (AC) joint injuries account for 9–12% of shoulder injuries, predominantly affecting young male athletes. While conservative treatment is established for Rockwood type I–II injuries and surgery is widely regarded as indicated for type V–VI, management of type III–IV injuries remains controversial. Biomechanical studies have shown superior results in combined reconstruction of the coracoclavicular and AC ligament complex, however clinical data is scarce. Therefore, the present study aimed to assess whether the addition of an acromioclavicular ligament reconstruction to an isolated coracoclavicular repair offers superior clinical and radiographic outcomes in the treatment of acute AC joint dislocations. **Methods:** A retrospective multicenter study was conducted on patients with Rockwood type III–VI AC joint injuries who underwent surgical treatment between 2019 and 2024. Patients were divided into two groups: isolated CC reconstruction (group I) and combined CC and AC ligament reconstruction (group II). Clinical outcome was assessed using patient-reported outcome measures (American Shoulder and Elbow Surgeons Score, Simple Shoulder Test, Single Assessment Numeric Evaluation, Visual Analogue Scale) and radiographic evaluations were performed regularly up to 6 months postoperatively. **Results:** Fifty-five patients (94.5% male, mean age 33.5 ± 10.9 years) were included in the present study. High patient satisfaction (group I: ASES 96.3 ± 7.9, SST 99.2 ± 3.3, SANE 95.3 ± 7.0; group II: ASES 95.8 ± 8.8, SST 96.8 ± 8.2, SANE 93.6 ± 12.8) was documented in both groups, but no significant differences were observed. The median coracoclavicular loosening ratio was 24.7% in the CC group and 32.6% in the CC and AC ligament reconstruction group (*p* = 0.830). Five complications occurred: two infections and three revision surgeries due to excessive secondary dislocations. **Conclusions:** Both surgical techniques demonstrated excellent clinical outcomes. In this study combined CC and AC ligament reconstruction did not yield superior clinical or radiological results compared to isolated coracoclavicular reconstruction. Our findings suggest that a routine AC ligament augmentation may not be necessary in all patients. Further randomized controlled trials are needed to validate these results.

## 1. Introduction

Acromioclavicular (AC) joint injuries are among the most common shoulder pathologies and are observed in approximately 9–12% of all shoulder injuries, particularly in young patients during sport activities, occurring five times more frequently in men than in women [1,2,3]. The most common trauma mechanism of AC joint injuries is a direct trauma to the shoulder with the arm in an adducted position, as is often documented in falls or impact during contact sports [1,4]. The shoulder girdle depends on the acromioclavicular joint for stability and mobility, which allows for overhead arm movement [5]. The AC joint is movable in all planes, however stabilization is guaranteed by the acromioclavicular as well as the coracoclavicular ligaments [2,3]. The acromioclavicular ligament complex consists of anterior, posterior, superior, and inferior ligaments whereas the coracoclavicular (CC) ligaments are formed by the trapezoid and conoid ligaments [2,3,6]. The AC ligament complex contributes to the horizontal stability of the AC joint, while the CC ligament complex mainly guarantees vertical stability [2,7]. Traditionally, classification of AC joint injuries is based on the Rockwood system, which categorizes these injuries into six distinctive types according to certain radiological changes [8]. Approaches for management of AC joint injuries are controversial, especially in grade ≥ III injuries [3,9]. However, there seems to be consensus for conservative treatment in grades I–II and surgical treatment is widely regarded as indicated for grades V–VI [2,9,10]. There is no standardized procedure on how to treat grade III–IV injuries. For type III AC joint injuries, the International Society of Arthroscopy, Knee Surgery and Orthopedic Sports Medicine [ISAKOS) provided a novel classification to subdivide these injuries into IIIa and IIIb lesions [11]. Type IIIa injuries are thought to be stable without the clavicle overriding on the cross-body adduction view, whereas type IIIb injuries manifest with horizontal instability and therapy-resistant scapular dysfunction [11]. Therefore, surgical treatment should be favored in type IIIb injuries, especially if initial conservative treatment fails. Haugaard et al., however, question the clinical relevance of this novel classification [12]. More than 150 different techniques have been described for surgical treatment of AC joint injuries [13]. Traditionally, an anatomic coracoclavicular reconstruction (ACCR) has been performed to reduce the AC joint without addressing the AC ligament complex [13]. However, isolated CC reconstruction may not produce optimal results when used for higher grade AC injuries, especially when horizontal instability is present [13]. Dyrna et al. postulated in 2018 that the AC capsule and its ligamentous complex are the major stabilizers against horizontal translation and posterior rotational forces [14]. A biomechanical study conducted concluded that combined stabilization of the AC capsule and CC ligaments showed the greatest capacity to restore native stability against translational and rotational loads [14]. Hislop et al. published similar results in a cadaveric study, highlighting the importance of the AC joint capsule and its associated soft tissues in order to provide horizontal stability [15].

To date there is a lack of clinical data comparing an isolated CC repair with a combined CC and AC ligament reconstruction. Therefore, this multicenter study aimed to assess whether the addition of an acromioclavicular ligament reconstruction to an isolated coracoclavicular repair offers superior clinical and radiographic outcomes in the treatment of acute AC joint dislocations.

## 2. Material and Methods

### 2.1. Patients and Study Design

This multicenter retrospective outcome study was approved by the respective local institutional review boards and complied with the guidelines of the Declaration of Helsinki. Retrospective chart reviews were performed at two urban trauma centers. All patients admitted from 2019 to 2024 were included if they (1) suffered an AC joint injury Rockwood III–VI, (2) were surgically treated, (3) were ≥18 years of age, and (4) had availability for follow-up of at least 6 months postoperatively. Patients suffering concomitant injuries were excluded from the present study.

### 2.2. Data Collection

Parameters collected included baseline characteristics (age, gender), dominant arm, extent of injury (Rockwood, ISAKOS), type of instability (acute, chronic), X-ray (stable, subluxation, dislocation), patient-reported outcome scores (American Shoulder and Elbow Surgeons Score, Simple Shoulder Test, Single Assessment Numeric Evaluation, Visual Analogue Scale). The primary outcome measure was patient-reported outcome scores at 6 months postoperatively.

### 2.3. Surgical Procedure

In both centers the procedures were performed by specialized shoulder surgeons. Patients were placed under general anesthesia before positioning them in a standard beach chair position. Two different fixation methods were applied. In group I, anatomic coracoclavicular reconstruction was performed in a mini-open technique utilizing one endobutton (AC-TightRope^®^, Arthrex, Naples, FL, USA) under direct fluoroscopic visualization without performing an additional AC ligament reconstruction.

In group II, arthroscopic-assisted reconstruction was performed using a similar technique as to described by Braun et al. [16]. Reconstruction of the coracoclavicular ligaments was achieved using a single endobutton (AC-TightRope^®^, Arthrex, Naples, FL, USA). After adequate reduction, a second horizontal skin incision was made over the AC joint and two holes were drilled, one in the lateral clavicle and one in the acromion, in an anterior-to-posterior direction with additional use of fluoroscopy. Consecutively, sutures (FiberTape^®^, Arthrex, Naples, FL, USA) were shuttled through and half hitches were tied down to create an X-shaped suture configuration. Patients in both groups were instructed to wear an arm sling for four weeks. Moreover, no abduction of the arm over 90° was allowed for 6 weeks postoperatively.

### 2.4. Radiographic Evaluation

Available x-rays of the shoulder were analyzed at two time points, immediately postoperatively as well as at the 6 month follow-up. For determination of vertical instability, x-ray evaluation was performed, similar to the technique Sahin et al. described [17]. The distance between the apex of the dorsal aspect of the coracoid and the corresponding inferior surface of the clavicle was quantified as the CC distance on postoperative radiographs as well as at the six-month postoperative interval. For evaluating AC distance, the anteromedial boarder of the acromion and the superolateral border of the clavicle were measured postoperatively and at the six-month follow-up on AP radiographs. The assessment of implant loosening and the loss of vertical stability at the time of follow-up was determined as the loosening ratio, expressed as a percentage.

Subsequently, the AC joint was graded as stable, subluxated, or dislocated based on the degree of translation (less than 50%, between 50% and 100%, or greater than 100%). Loosening ratio over 100% was considered as failure. All images were processed with PACS (Picture Archiving and Communication System), and one blinded investigator (F.P.) performed all measurements.

### 2.5. Statistical Analysis

All statistical analyses were performed using IBM SPSS^®^ Statistics for Mac, version 26.0 (IBM Corp., Armonk, NY, USA).

The Kolmogorov–Smirnov test was used to assess whether the parameters followed a normal distribution. Normally distributed parameters are presented as mean ± standard deviation, whereas non-normally distributed parameters are reported as median. Frequency counts and percentages characterize categorical data. These were analyzed using the χ^2^ test. Mann–Whitney U tests were used to compare independent groups. The Student’s *t* test was used to compare the means between two groups. Spearman’s rank correlation coefficient was used to measure the strength and direction of association between patient-reported outcome scores. Stacked bar charts were established to visualize results. In general, a *p*-value < 0.05 was considered significant.

## 3. Results

### 3.1. Study Population

The present study consisted of 55 patients (94.5% male, mean age 33.5 ± 10.9 years, range: 18–54 years) in total. As listed in Table 1, the CC reconstruction group consisted of 33 patients (mean age 32.2 ± 10.8 years, 93.9% male), whereas the combined CC and AC ligament reconstruction group consisted of 22 patients (mean age 35.2 ± 11.3 years, 95.5% male). There were no significant differences regarding gender and age distribution between both groups.

### 3.2. Patient-Reported Outcome Scores

As reported in Table 2, all documented patient-reported outcome scores were highly satisfactory, however no significant differences were detected between the two fixation methods (Table 1). Spearman’s rank correlation coefficient furthermore revealed a significant correlation between ASES and SST (*p* < 0.001), ASES and SANE (*p* < 0.001), and SANE and SST (*p* < 0.001). There was a significant overall decline in the Visual Analogue Scale (VAS) comparing preoperative to postoperative results (CC: 6.1 ± 2.7 pre-OP vs. 0.5 ± 1.0 post-OP, *p* < 0.001; CC + AC: 6.2 ± 1.1 pre-OP vs. 1.0 ± 1.5 post-OP, *p* < 0.001).

### 3.3. Radiographic Evaluation

**Figure 1:**
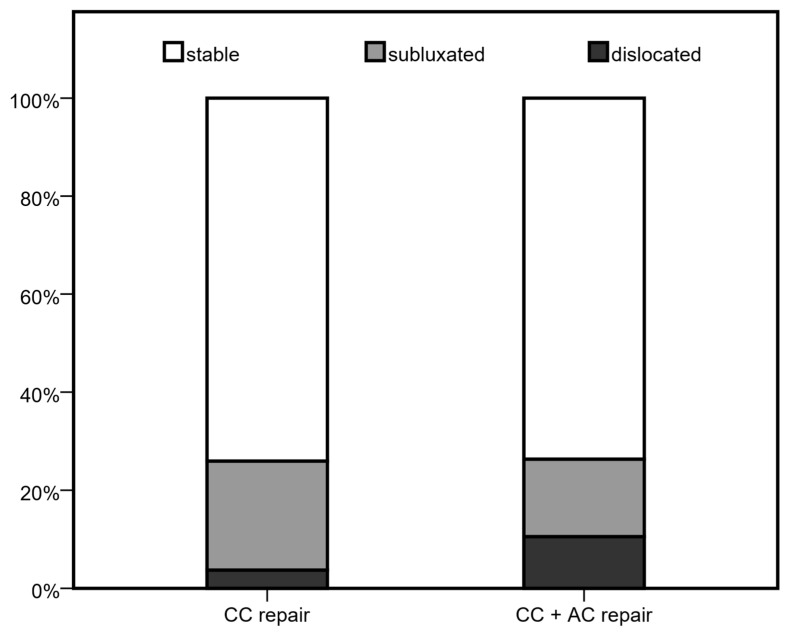
AC joint position at 6 month follow-up.

**Figure 2:**
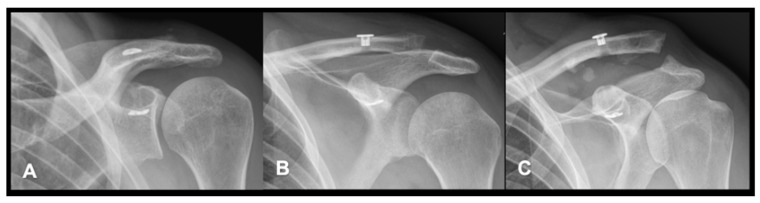
Display of AC joints ((**A**): stable, (**B**): subluxated, (**C**): dislocated).

**Figure 3:**
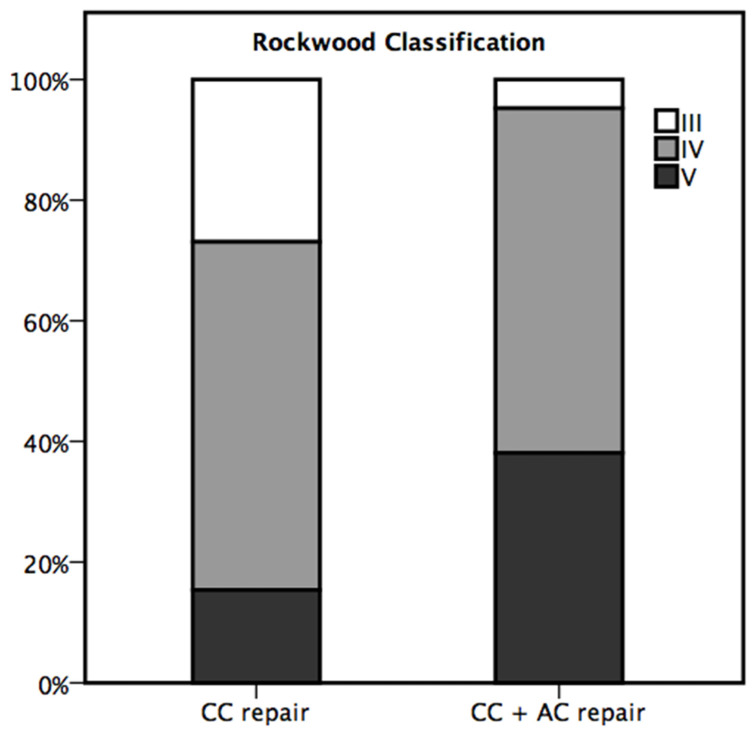
Distribution of AC joint injuries following trauma according to Rockwood et al. [8].

A total of 46 x-rays of the shoulder were available for evaluation at 6 months of follow-up. As seen in Table 3, stable AC joints were documented in 34 patients (73.9%), whereas 9 (19.6%) AC joints were subluxated and 3 (6.6%) were dislocated. Figure 1 and Figure 2 illustrate the three different joint positions and their documented distribution. In the present study combined CC and AC ligament reconstruction was more frequently observed in higher grade AC joint dislocations (*p* = 0.020) compared to isolated coracoclavicular reconstruction. Figure 3 displays the distribution of AC joint injuries following trauma according to the classification by Rockwood et al. [8]. We found a median coracoclavicular loosening ratio of 24.7% in the CC group and 32.6% in the CC and AC ligament reconstruction group (*p* = 0.830).

### 3.4. Intervention-Specific Outcomes

As seen in Table 4, the mean duration of surgery was reported with 80.2 min in the CC group, whereas a mean of 90.6 min was reported in the CC and AC ligament reconstruction group (*p* = 0.119). The mean time to surgery after trauma was documented with 11.2 days in the CC group versus 10.0 days in the CC and AC reconstruction group (*p* = 0.378). There was no association between the time to surgery following trauma and patient-reported outcome scores in the present study. In the CC and AC reconstruction group, four (18.2%) concomitant procedures were observed compared to zero concomitant procedures in the isolated CC repair group (*p* = 0.021). These were labral repairs in three cases as well as one reduction of a fractured greater tubercle. Five complications (9.1%) were documented in total. One infection, as well as one fracture of the coracoid, was observed in the CC group, whereas one infection and two revision surgeries due to excessive secondary dislocations were documented in the CC and AC reconstruction group.

## 4. Discussion

This multicenter retrospective study aimed to assess whether the addition of an acromioclavicular ligament reconstruction to an isolated coracoclavicular repair provides superior clinical and radiographic outcomes in the treatment of acute AC joint dislocations. The data suggest that while both surgical techniques yielded satisfactory results in terms of pain reduction (VAS) and patient-reported outcome scores (ASES, SST, SANE), no significant clinical benefit at the 6 month follow-up was observed with the addition of AC ligament reconstruction. Moreover, radiographic analysis revealed no improvement in vertical joint stability in the combined AC and CC reconstruction group.

The standard surgical treatment for dislocated AC joints, particularly in Rockwood grade III–V injuries, has traditionally been solitary anatomic coracoclavicular ligament reconstruction [2]. However, recent literature has increasingly emphasized the importance of restoring horizontal stability, which is primarily maintained by the AC capsule and its ligamentous structures [2,18,19]. In this context, Dyrna et al. demonstrated that isolated CC reconstruction failed to restore the physiological kinematics of the AC joint and that additional AC ligament reconstruction significantly improved stability in cadaveric models [14]. However, clinical validation of these biomechanical assumptions remains scarce. In the present study, however, we were unable to detect superior clinical or radiological results when additional AC reconstruction was performed. This contrasts with prior biomechanical evidence suggesting that isolated CC reconstruction may fail to address horizontal instability adequately. A study by Sahin et al. also found no significant association of an additional AC reconstruction with beneficial clinical outcomes [17]. Despite the study’s limited sample size of only 19 patients, their findings and ours indicate that there is a disparity between the biomechanical and clinical results [17].

One possible explanation for this disparity between clinical and biomechanical findings could be the inability of the applied scores to detect modest changes, especially the horizontal translation. We hypothesize that patient-reported outcome measures as used in the present study could plateau in young and active patients, potentially masking minor functional deficits. Furthermore, Baumgarten et al. showed a high correlation of the American Shoulder and Elbow Surgeons score with the Simple Shoulder Test [20], which validates the results of the present study.

Notably, combined CC and AC ligament reconstruction was significantly more prevalent among patients presenting with higher grade AC joint dislocations in the present study. Our results reflect current concepts of treating horizontal instability with an additional acromioclavicular capsule repair, especially in higher grade AC joint injuries [2,14,21]. One reason for not observing significant clinical differences between the two fixation methods might be the surgeon’s preference to restore AC joint stability with an additional AC ligament reconstruction in higher grade AC joint injuries.

In the present study, the complication rate was relatively low and evenly distributed between groups, contradicting previous findings with documented complication rates of up to 27% [22,23]. Patients were included in this study from 2019 onwards, thus we hypothesize that the treating surgeons were experienced in handling modern fixation systems such as the AC-TightRope^®^. As such, we conclude that these fixation devices are safe and effective for both isolated and combined procedures.

Although the difference in operative time between the two fixation methods did not reach statistical significance, there is a trend toward shorter surgical durations in the isolated coracoclavicular repair group. Independent of the performed surgery or specialty, it is well documented that prolonged operative time is associated with an increase in the risk of overall complications [24,25], even correlating with a decrease in functional scores following shoulder surgeries [26]. Furthermore, the positive correlation between surgery time and mean costs in the operating room adds a socioeconomic aspect to the longer procedures [27]. If future randomized controlled trials with larger patient cohorts validate these trends, we advocate for a preference for a less time-consuming surgery, provided it demonstrates non-inferiority in clinical outcomes.

Moreover, we want to highlight that arthroscopic-assisted reconstruction of the CC and AC ligament complex as conducted in group II led to diagnosis of labral tears in three cases, which were subsequently treated. This finding aligns with numerous recent publications where intraarticular comorbidities such as lesions of the long head of the biceps tendon or SLAP lesions (superior labral anterior posterior lesions) have been reported in up to 27% of AC joint dislocations [2,28,29]. The authors therefore conclude that the diagnosis and immediate treatment of concomitant pathologies is an extraordinary advantage in the arthroscopic management of AC joint injuries in comparison to the conventional mini-open technique.

## 5. Limitations

The present study is subject to several limitations. First, the relatively small sample size and the retrospective design conducted over an extended time period may limit the generalizability of the findings. Second, the inherent heterogeneity of AC joint injuries complicates direct comparisons between treatment groups. Additionally, the assessment of gender-specific variations was not possible in the current investigation owing to the limited sample size. Moreover, we want to highlight that the CC and AC group had a significantly higher proportion of Rockwood V injuries, which represents a potential major confounder. However, conducting a subgroup analysis of Rockwood V injuries was not reasonable due to the limited sample size. Finally, the lack of standardized radiographic imaging prevented the evaluation of horizontal instability of the AC joint.

## 6. Conclusions

The present study demonstrated excellent patient satisfaction in isolated anatomical coracoclavicular reconstruction as well as in combined coracoclavicular and acromioclavicular ligament repair in acute AC joint dislocations. To our knowledge, this is the only multicenter study of its kind. The authors conclude that isolated CC reconstruction offers comparable clinical outcomes to combined CC and AC ligament reconstruction. While biomechanical evidence suggests potential benefits of additional acromioclavicular repair, the clinical superiority of such an approach remains unproven. Our findings suggest that a standardized AC ligament augmentation may not be necessary in all patients. Future randomized controlled trials with larger patient cohorts are essential to verify if clinical outcomes differ in patients with additional acromioclavicular reconstruction after suffering an AC joint injury.

## Figures and Tables

**Table 1:** Patient characteristics.

	CC	CC + AC	*p*-Value
Number (*n*)	33	22	-
Males:females (*n*)	31:2	21:1	1.000
Age (years), M ± SD	32.2 ± 10.8	35.2 ± 11.3	0.331
Right Shoulder, *n* (%)	13 (39.4)	13 (59.1)	0.152

**Table 2:** Patient-Reported Outcome Scores.

	CC	CC + AC	*p*-Value
ASES, M ± SD	96.3 ± 7.9	95.8 ± 8.8	0.828
SST, M ± SD	99.2 ± 3.3	96.8 ± 8.2	0.210
SANE, M ± SD	95.3 ± 7.0	93.6 ± 12.8	0.589
VAS pre-OP, M ± SD	6.1 ± 2.7	6.2 ± 1.1	0.846
VAS post-OP, M ± SD	0.5 ± 1.0	1.0 ± 1.5	0.129

**Table 3:** Radiographic Evaluation.

	CC	CC + AC	*p*-Value
Rockwood/ISAKOS			-
III, *n* (%)	7 (21.2)	1 (4.5)	**0.020**
IIIa, *n*	1/7	0/1	
IIIb, *n*	6/7	1/1	
IV, *n* (%)	15 (45.5)	12 (54.5)	
V, *n* (%)	4 (12.1)	8 (36.4)	
VI, *n* (%)	0 (0.0)	0 (0.0)	
Vertical stability			-
stable, *n* (%)	20 (74.1)	14 (73.7)	0.597
subluxation, *n* (%)	6 (22.2)	3 (15.8)	
dislocation, *n* (%)	1 (3.7)	2 (10.5)	
CC-Loosening ratio, (%) Md	24.7 (*n* = 27)	32.6 (*n* = 19)	0.830
AC-Loosening ratio, (%) Md	72.6 (*n* = 27)	106.5 (*n* = 19)	0.567

**Table 4:** Intervention-specific outcomes.

	CC	CC + AC	*p*-Value
Duration of surgery, min M ± SD	80.2 ± 23.2	90.6 ± 24.8	0.119
Time to surgery, (days), M ± SD	11.2 ± 5.4	10.0 ± 3.5	0.378
Concomitant procedure, *n* (%)	0 (0)	4 (18.2)	0.021
Complications, *n* (%)	2 (6.1)	3 (13.6)	0.379
Chronic instability, *n* (%)	3 (5.2%)		-

## Data Availability

The analyzed dataset in this study is available from the first author upon reasonable request.
